# Effect, economic and process evaluation of a mental health return to work intervention: study design: Fiona Starke

**DOI:** 10.1093/eurpub/ckac130.211

**Published:** 2022-10-25

**Authors:** F Starke, A de Rijk, U Wegewitz

**Affiliations:** 1 Division 3 Work and Health, Federal Institute for Occupational Health & Safety, Berlin, Germany; 2 Department of Social Medicine, Maastricht University, Maastricht, Netherlands

## Abstract

**Background:**

In Germany, the sickness absence duration for mental disorders lasts on average more than twice as long as for all other diseases. Interventions that link medical-therapeutic approaches with organisation-directed methods to improve a sustainable return to work (RTW) have hardly been evaluated. The aim is to present the study design, which evaluates the (cost-)effectiveness and feasibility of an 18-month intensified RTW aftercare delivered by German psychiatric outpatient clinics to employees with mental disorders.

**Methods:**

A two-arm multicentre randomised controlled study is carried out. Sick-listed employees with mental disorders are recruited within psychiatric hospitals (n = 506). Besides care as usual, the intervention group receives a multimodal treatment consisting of individual RTW-support, RTW-aftercare groups, and web-based aftercare. At five time points over a 24-month observation period, quantitative effectiveness and health economic evaluation data from a societal perspective will be obtained. Intervention fidelity, reach, context, satisfaction and dose delivered will be examined in a multi-method process evaluation. A qualitative evaluation using pre-and post-interviews from a sub-sample (n = 30) will complement the effectiveness and process evaluation findings.

**Results:**

Recruitment is currently ongoing. The intervention is expected to improve the employeés achievement of a sustainable RTW, defined as the 12-month remain and participation at work without having sickness absence periods of > 6 weeks (after RTW). It is hypothesized that the intervention is preferable in terms of costs compared to care as usual. The interviews allow for a better understanding of subjective experiences with the intervention.

**Discussion:**

This study evaluates the (cost-)effectiveness and implementation process of an intensified RTW aftercare. If successfully implemented and evaluated, it might be adopted as standard care for employees with mental disorders in Germany.

**Key messages:**

- A comprehensive evaluation of a RTW-intervention is needed.
- The proposed design includes an effectiveness, process and health economic evaluation. It has a unique scope and follow-up length.

